# A compressed octa­hedral cobalt(II) complex in the crystal structure of di­aqua­[6,6′-sulfanediylbis(2,2′-bi­pyridine)]cobalt(II) dinitrate

**DOI:** 10.1107/S2056989017008428

**Published:** 2017-06-13

**Authors:** Guo-Ling Li, Osamu Sato

**Affiliations:** a Institute for Materials Chemistry and Engineering, Kyushu University, 744 Motoka, Nishi-ku, Fukuoka 819-0395, Japan

**Keywords:** crystal structure, cobalt complex, 2,2′-bi­pyridine derivative, compressed octa­hedral geometry, hydrogen bond

## Abstract

The asymmetric unit of the title salt, [Co(C_20_H_14_N_4_S)(H_2_O)_2_](NO_3_)_2_, comprises a [Co(C_20_H_14_N_4_S)(H_2_O)_2_]^2+^ cation and two NO_3_^−^ anions. In the complex, [Co(C_20_H_14_N_4_S)(H_2_O)_2_]^2+^ cation, the tetra­dentate 6,6′-sulfanediylbis(2,2′-bi­pyridine) ligand coordinates to the Co^II^ cation in the equatorial positions, while two water mol­ecules occupy the axial positions, forming a compressed octa­hedral CoN_4_O_2_ coordination sphere. The NO_3_^−^ anions are linked to the [Co(C_20_H_14_N_4_S)(H_2_O)_2_]^2+^ cations *via* O—H⋯O hydrogen bonds, yielding a layered arrangement parallel to (001).

## Abstract 2

The [Co(C_20_H_14_N_4_S)(H_2_O)_2_]^2+^ cation in the title compound possesses pseudo-*C*_2v_ point-group symmetry.

## Chemical context   

The control of the mol­ecular structure of coordination compounds is an important task in crystal engineering. It is well known that organic ligands play a significant role in determining the crystal structure of coordination complexes. For example, bidentate 2,2′-bi­pyridine or its derivatives are common ligands that can be employed to assemble functional compounds (Zhang *et al.*, 2014 ▸; Kamdar *et al.*, 2016 ▸; Pal *et al.*, 2014 ▸). Linking two 2,2′-bi­pyridine units through a suitable atom leads to a tetra­dentate ligand (Knight *et al.*, 2010 ▸) and, more importantly, the distance of the two 2,2′-bi­pyridine moieties can then be controlled by the type and size of the bridging atom. As a consequence, the coordination geometry of the metal cation can be affected.
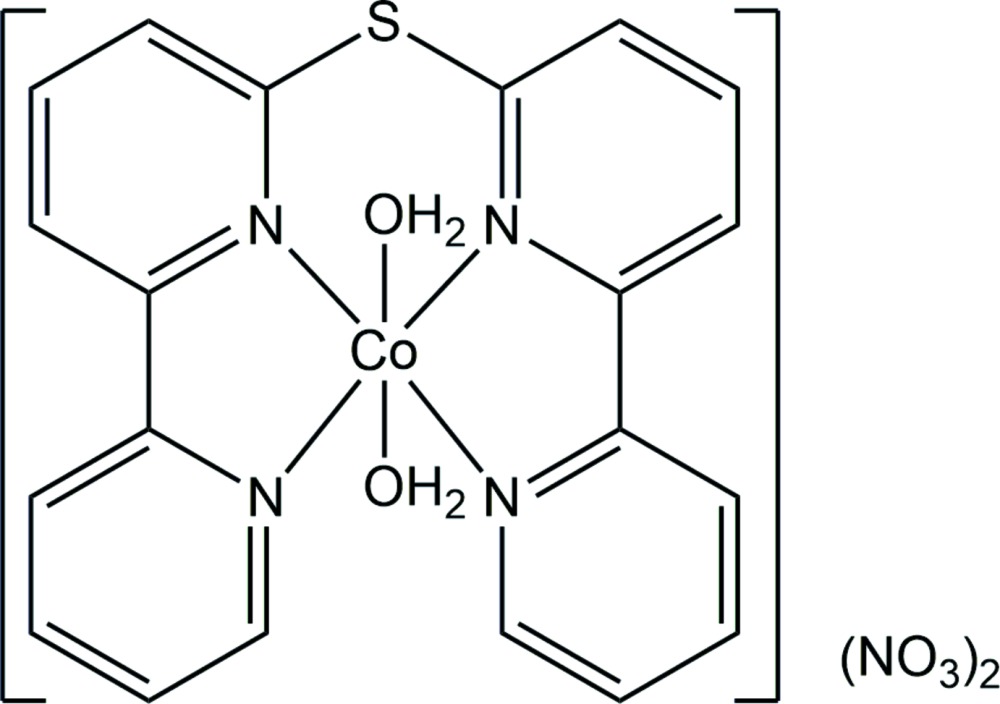


Recently, we obtained the title salt, [Co(C_20_H_14_N_4_S)(H_2_O)_2_](NO_3_)_2_, using a tetra­dentate ligand in which two 2,2′-bi­pyridine moieties are linked by a sulfur atom. Herein, we report the crystal structure of this cobalt complex.

## Structural commentary   

**Figure 1:**
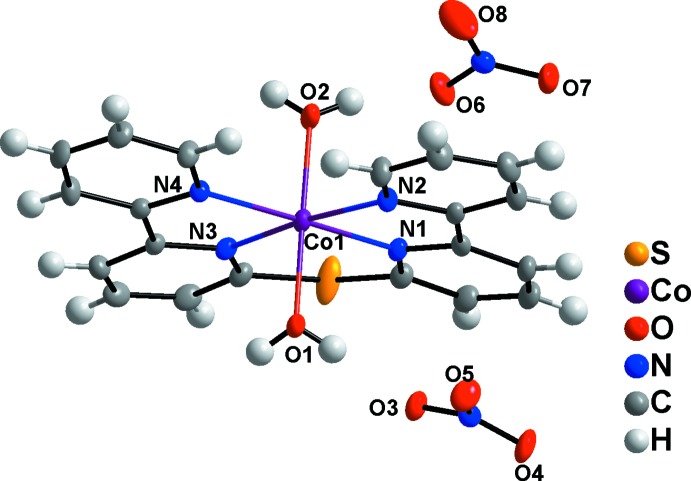
The structures of the mol­ecular entities in the structure of the title salt. Displacement ellipsoids are drawn at the 50% probability level.

The asymmetric unit of the title salt (Fig. 1 ▸) is composed of a [Co(C_20_H_14_N_4_S)(H_2_O)_2_]^2+^ cation and two NO_3_^−^ anions. The cobalt(II) atom of the complex [Co(C_20_H_14_N_4_S)(H_2_O)_2_]^2+^ cation features a compressed octa­hedral CoN_4_O_2_ coordination sphere with the N atoms of the tetra­dentate ligand in equatorial positions and two water mol­ecules located at the *trans* axial sites. The corresponding Co—O bond lengths are 2.0444 (18) Å and 2.0821 (17) Å, which are obviously shorter than the equatorial Co—N bond lengths [2.1213 (18) −2.1574 (18) Å]. These coordination bond lengths indicate that the Co^II^ cation is in a high-spin state at 123 K, comparable with other high-spin Co^II^ complexes (Li *et al.*, 2016 ▸; Knight *et al.*, 2010 ▸; Suckert *et al.*, 2017 ▸; Zhong *et al.*, 2008 ▸; Hathwar *et al.*, 2017 ▸). The O—Co—O angle is almost linear at 178.59 (7)°. The four equatorial N atoms and the Co^II^ cation are approximately coplanar, with the largest deviation from the least-squares plane being 0.039 Å for N3.

In a similar Co^II^ complex with the 6,6′-sulfanediylbis(2,2′-bi­pyridine) ligand replaced by the tetra­dentate ligand bis­(2,2′-bipyrid-6′-yl)ketone (Knight *et al.*, 2010 ▸), the Co^II^ cation is slightly convex (0.098 Å) from the plane formed through four coordination N atoms. The Co—O bond lengths of the two axial sites are significantly different at 2.075 (4) Å for that in the convex site and 2.118 (4) Å for that in the concave site. The corresponding O—Co—O bond angle deviates more distinctly from linearity with a value of 172.46 (17)°. The structural differences between the title complex and the similar reported compound are ascribed to the bridging atom between the two 2,2′-bi­pyridine moieties, *i.e*. an S atom in the title complex *versus* a C atom of a keto group in the related compound. The bridging bonds [C—S: 1.761 (2) and 1.764 (2) Å] of the title complex are longer than those [C—C: 1.496 (10) and 1.500 (10) Å] in the related complex.

## Supra­molecular features   

**Figure 2:**
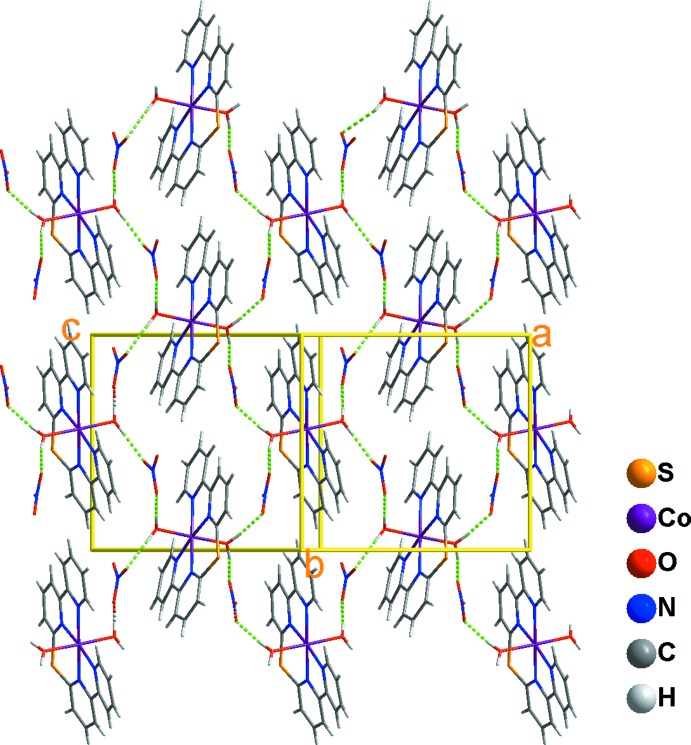
The layer structure in the title salt formed through hydrogen bonds (green dotted lines) between complex cations and nitrate anions.

The coordinating water mol­ecules act as proton donors, forming O—H⋯O hydrogen bonds with the NO_3_^−^ anions and leading to an extended layer structure parallel to (001) for the title complex (Fig. 2 ▸). For these hydrogen bonds, the O⋯O distances are in the range of 2.688 (2)–2.789 (2) Å, indicating they are of medium strength (Table 1 ▸), and are comparable with other hydrogen bonds formed between coordinating water mol­ecules and NO_3_^−^ anions (Kurdziel *et al.*, 2000 ▸; Kunz *et al.*, 2007 ▸; Wang *et al.*, 2012 ▸). There are no inter­molecular π–π inter­actions in the mol­ecular packing of the title complex.

## Synthesis and crystallization   

The ligand 6,6′-sulfanediylbis(2,2′-bi­pyridine) was synthesized by a method analogous to that for the preparation of 2,2′-sulfanediylbis(1,10-phenanthroline) (Krapcho *et al.*, 2007 ▸). The title complex was obtained as follows: An ethano­lic solution (10 ml) of Co^II^(NO_3_)_2_·6H_2_O (29.1 mg, 0.1 mmol) was added to a ethano­lic solution (10 ml) of 6,6′-sulfane­diylbis(2,2′-bi­pyridine) (34.4 mg, 0.1 mmol), which afforded a light-yellow solution, which was stored at ambient conditions. Yellow crystals of the title compound were obtained by slow evaporation of the solvent, yield: *ca*. 50%.

## Refinement   

Crystal data, data collection and structure refinement details are summarized in Table 2 ▸. All hydrogen atoms bound to carbon atoms were placed geometrically, with C—H = 0.93 Å and with *U*_iso_(H) = 1.2*U*_eq_(C). The hydrogen atoms of water mol­ecules were found from difference-Fourier maps and their O—H bond lengths were normalized to 0.82 Å and refined with a common *U*_iso_(H) parameter.

## Supplementary Material

Crystal structure: contains datablock(s) I. DOI: 10.1107/S2056989017008428/wm5395sup1.cif

Structure factors: contains datablock(s) I. DOI: 10.1107/S2056989017008428/wm5395Isup2.hkl

CCDC reference: 1554624

Additional supporting information:  crystallographic information; 3D view; checkCIF report

## Figures and Tables

**Table 1:** Hydrogen-bond geometry (Å, °)

*D*—H⋯*A*	*D*—H	H⋯*A*	*D*⋯*A*	*D*—H⋯*A*
O2—H2*WB*⋯O7^i^	0.80 (2)	2.02 (2)	2.766 (3)	155 (2)
O2—H2*WA*⋯O6	0.84 (2)	1.88 (2)	2.698 (2)	168 (3)
O1—H1*WB*⋯O5^ii^	0.83 (2)	1.96 (2)	2.789 (2)	179 (3)
O1—H1*WA*⋯O3	0.80 (2)	1.89 (2)	2.688 (2)	174 (3)

Symmetry codes: (i) 
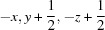
; (ii) 
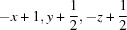
.

**Table 2:** Experimental details

Crystal data	
Chemical formula	[Co(C_20_H_14_N_4_S)(H_2_O)_2_](NO_3_)_2_
*M*_r_	561.39
Crystal system, space group	Monoclinic, *P*2_1_/*c*
Temperature (K)	123
*a*, *b*, *c* (Å)	13.412 (3), 11.421 (2), 15.441 (3)
β (°)	110.50 (3)
*V* (Å^3^)	2215.4 (9)
*Z*	4
Radiation type	Mo *K*α
μ (mm^−1^)	0.93
Crystal size (mm)	0.15 × 0.14 × 0.11

Data collection	
Diffractometer	Rigaku Saturn724
Absorption correction	Multi-scan (*CrystalClear*; Rigaku, 2008 ▸)
*T*_min_, *T*_max_	0.893, 1.000
No. of measured, independent and observed [*I* > 2σ(*I*)] reflections	17851, 5045, 3984
*R*_int_	0.044
(sin θ/λ)_max_ (Å^−1^)	0.649

Refinement	
*R*[*F* ^2^ > 2σ(*F* ^2^)], *wR*(*F* ^2^), *S*	0.042, 0.090, 1.06
No. of reflections	5045
No. of parameters	337
No. of restraints	4
H-atom treatment	H atoms treated by a mixture of independent and constrained refinement
Δρ_max_, Δρ_min_ (e Å^−3^)	0.46, −0.42

Computer programs: *CrystalClear* (Rigaku, 2008 ▸), *SHELXS97* and *SHELXTL* (Sheldrick, 2008 ▸), *SHELXL2014* (Sheldrick, 2015 ▸) and *publCIF* (Westrip, 2010 ▸).

## supplementary crystallographic information

### Crystal data

**Table:**

[Co(C_20_H_14_N_4_S)(H_2_O)_2_](NO_3_)_2_	*F*(000) = 1148
*M**_r_* = 561.39	*D*_x_ = 1.683 Mg m^−^^3^
Monoclinic, *P*2_1_/*c*	Mo *K*α radiation, λ = 0.71073 Å
*a* = 13.412 (3) Å	Cell parameters from 5682 reflections
*b* = 11.421 (2) Å	θ = 3.1–27.5°
*c* = 15.441 (3) Å	µ = 0.93 mm^−^^1^
β = 110.50 (3)°	*T* = 123 K
*V* = 2215.4 (9) Å^3^	Block, yellow
*Z* = 4	0.15 × 0.14 × 0.11 mm

### Data collection

**Table:**

Rigaku Saturn724 diffractometer	5045 independent reflections
Radiation source: Rotating Anode	3984 reflections with *I* > 2σ(*I*)
Detector resolution: 28.5714 pixels mm^-1^	*R*_int_ = 0.044
dtprofit.ref scans	θ_max_ = 27.5°, θ_min_ = 3.1°
Absorption correction: multi-scan (*CrystalClear*; Rigaku, 2008)	*h* = −17→17
*T*_min_ = 0.893, *T*_max_ = 1.000	*k* = −14→10
17851 measured reflections	*l* = −20→19

### Refinement

**Table:**

Refinement on *F*^2^	4 restraints
Least-squares matrix: full	Hydrogen site location: mixed
*R*[*F*^2^ > 2σ(*F*^2^)] = 0.042	H atoms treated by a mixture of independent and constrained refinement
*wR*(*F*^2^) = 0.090	*w* = 1/[σ^2^(*F*_o_^2^) + (0.040*P*)^2^ + 0.524*P*] where *P* = (*F*_o_^2^ + 2*F*_c_^2^)/3
*S* = 1.06	(Δ/σ)_max_ = 0.001
5045 reflections	Δρ_max_ = 0.46 e Å^−^^3^
337 parameters	Δρ_min_ = −0.42 e Å^−^^3^

### Special details

**Table:**

Geometry. All esds (except the esd in the dihedral angle between two l.s. planes) are estimated using the full covariance matrix. The cell esds are taken into account individually in the estimation of esds in distances, angles and torsion angles; correlations between esds in cell parameters are only used when they are defined by crystal symmetry. An approximate (isotropic) treatment of cell esds is used for estimating esds involving l.s. planes.

### Fractional atomic coordinates and isotropic or equivalent isotropic displacement parameters (Å^2^)

**Table:**

	*x*	*y*	*z*	*U*_iso_*/*U*_eq_	
S2	0.34784 (7)	0.53580 (6)	0.48067 (4)	0.0421 (2)	
Co1	0.24302 (2)	0.43855 (2)	0.24018 (2)	0.01606 (9)	
O1	0.39231 (13)	0.40239 (14)	0.23348 (10)	0.0187 (3)	
O2	0.09507 (14)	0.47032 (15)	0.24506 (13)	0.0285 (4)	
O3	0.50179 (13)	0.23807 (13)	0.35394 (10)	0.0236 (4)	
O4	0.54960 (14)	0.06402 (14)	0.40822 (11)	0.0300 (4)	
O6	0.05056 (14)	0.33139 (14)	0.36815 (11)	0.0292 (4)	
O5	0.47102 (14)	0.08860 (15)	0.26072 (10)	0.0297 (4)	
O7	0.02144 (15)	0.14605 (15)	0.36918 (13)	0.0393 (5)	
O8	−0.06083 (17)	0.2472 (2)	0.24938 (13)	0.0593 (7)	
N5	0.50719 (15)	0.12943 (16)	0.34121 (13)	0.0198 (4)	
N6	0.00328 (15)	0.24137 (17)	0.32853 (13)	0.0212 (4)	
N2	0.19761 (15)	0.26188 (16)	0.19352 (12)	0.0186 (4)	
N4	0.20317 (14)	0.55097 (15)	0.12099 (12)	0.0166 (4)	
N3	0.29863 (14)	0.60458 (15)	0.29886 (12)	0.0172 (4)	
N1	0.28384 (14)	0.34360 (15)	0.36610 (12)	0.0175 (4)	
C7	0.18108 (19)	0.0673 (2)	0.24432 (16)	0.0238 (5)	
H7A	0.1923	0.0149	0.2929	0.029*	
C8	0.1315 (2)	0.0304 (2)	0.15461 (17)	0.0276 (6)	
H8A	0.1085	−0.0466	0.1419	0.033*	
C6	0.21437 (17)	0.18271 (19)	0.26220 (14)	0.0176 (5)	
C4	0.30035 (18)	0.1515 (2)	0.43295 (15)	0.0229 (5)	
H4A	0.2909	0.0711	0.4245	0.027*	
C10	0.15035 (19)	0.2232 (2)	0.10636 (16)	0.0250 (5)	
H10A	0.1398	0.2763	0.0583	0.030*	
C9	0.1167 (2)	0.1098 (2)	0.08414 (16)	0.0280 (6)	
H9A	0.0846	0.0871	0.0227	0.034*	
C5	0.26838 (17)	0.22627 (19)	0.35760 (15)	0.0179 (5)	
C2	0.36036 (19)	0.3163 (2)	0.53060 (16)	0.0242 (5)	
H2A	0.3905	0.3495	0.5890	0.029*	
C1	0.32874 (18)	0.3862 (2)	0.45197 (15)	0.0214 (5)	
C3	0.34627 (19)	0.1974 (2)	0.52047 (16)	0.0262 (5)	
H3A	0.3674	0.1486	0.5719	0.031*	
C14	0.33856 (18)	0.8009 (2)	0.26565 (16)	0.0220 (5)	
H14A	0.3370	0.8578	0.2221	0.026*	
C16	0.23741 (17)	0.66245 (19)	0.13919 (14)	0.0175 (5)	
C19	0.11790 (19)	0.6048 (2)	−0.03804 (15)	0.0236 (5)	
H19A	0.0759	0.5828	−0.0977	0.028*	
C17	0.21498 (19)	0.7466 (2)	0.07011 (15)	0.0232 (5)	
H17A	0.2403	0.8226	0.0844	0.028*	
C20	0.14367 (18)	0.5253 (2)	0.03312 (15)	0.0224 (5)	
H20A	0.1184	0.4492	0.0197	0.027*	
C11	0.34248 (18)	0.63032 (19)	0.38876 (15)	0.0202 (5)	
C15	0.29568 (17)	0.69114 (18)	0.23791 (14)	0.0166 (5)	
C18	0.15527 (19)	0.7174 (2)	−0.01954 (15)	0.0242 (5)	
H18A	0.1405	0.7726	−0.0666	0.029*	
C13	0.38377 (18)	0.8247 (2)	0.35920 (16)	0.0249 (5)	
H13A	0.4132	0.8979	0.3793	0.030*	
C12	0.38477 (19)	0.7396 (2)	0.42191 (16)	0.0240 (5)	
H12B	0.4130	0.7544	0.4851	0.029*	
H1WA	0.4237 (19)	0.3504 (18)	0.2663 (15)	0.029*	
H1WB	0.4339 (18)	0.4572 (18)	0.2351 (17)	0.029*	
H2WA	0.072 (2)	0.430 (2)	0.2791 (15)	0.029*	
H2WB	0.0665 (19)	0.5317 (17)	0.2273 (17)	0.029*	

### Atomic displacement parameters (Å^2^)

**Table:**

	*U*^11^	*U*^22^	*U*^33^	*U*^12^	*U*^13^	*U*^23^
S2	0.0871 (6)	0.0210 (3)	0.0148 (3)	0.0028 (4)	0.0135 (3)	−0.0002 (3)
Co1	0.02039 (16)	0.01296 (16)	0.01403 (17)	0.00085 (12)	0.00502 (12)	0.00167 (12)
O1	0.0214 (9)	0.0152 (8)	0.0188 (8)	0.0018 (7)	0.0061 (7)	0.0044 (6)
O2	0.0270 (10)	0.0227 (10)	0.0398 (11)	0.0072 (8)	0.0165 (8)	0.0158 (8)
O3	0.0325 (10)	0.0146 (8)	0.0223 (9)	0.0043 (7)	0.0077 (7)	0.0010 (7)
O4	0.0419 (11)	0.0178 (9)	0.0241 (9)	0.0056 (8)	0.0038 (8)	0.0073 (7)
O6	0.0407 (11)	0.0191 (9)	0.0325 (9)	−0.0100 (8)	0.0186 (8)	−0.0077 (7)
O5	0.0388 (11)	0.0276 (10)	0.0172 (9)	0.0020 (8)	0.0030 (8)	−0.0066 (7)
O7	0.0359 (11)	0.0206 (9)	0.0598 (12)	0.0024 (8)	0.0148 (9)	0.0163 (9)
O8	0.0515 (14)	0.0879 (18)	0.0237 (11)	−0.0330 (13)	−0.0057 (10)	0.0093 (11)
N5	0.0200 (10)	0.0177 (10)	0.0228 (10)	−0.0004 (8)	0.0087 (8)	−0.0011 (8)
N6	0.0193 (10)	0.0231 (11)	0.0231 (11)	0.0000 (8)	0.0097 (9)	0.0003 (9)
N2	0.0214 (10)	0.0158 (9)	0.0173 (10)	−0.0014 (8)	0.0052 (8)	0.0008 (8)
N4	0.0178 (9)	0.0159 (9)	0.0149 (9)	−0.0006 (8)	0.0041 (7)	0.0011 (7)
N3	0.0192 (9)	0.0161 (9)	0.0150 (9)	0.0023 (8)	0.0045 (8)	−0.0006 (8)
N1	0.0206 (10)	0.0162 (10)	0.0164 (9)	0.0025 (8)	0.0075 (8)	0.0027 (8)
C7	0.0293 (13)	0.0169 (12)	0.0281 (13)	−0.0005 (10)	0.0139 (11)	0.0028 (10)
C8	0.0337 (14)	0.0179 (12)	0.0329 (14)	−0.0062 (11)	0.0138 (11)	−0.0031 (10)
C6	0.0184 (11)	0.0155 (11)	0.0213 (12)	0.0029 (9)	0.0102 (9)	0.0037 (9)
C4	0.0254 (12)	0.0184 (12)	0.0254 (13)	0.0023 (10)	0.0095 (10)	0.0076 (10)
C10	0.0328 (14)	0.0204 (12)	0.0197 (12)	−0.0018 (11)	0.0064 (10)	0.0029 (10)
C9	0.0340 (14)	0.0250 (13)	0.0227 (13)	−0.0054 (11)	0.0071 (11)	−0.0035 (10)
C5	0.0188 (11)	0.0164 (11)	0.0219 (12)	0.0012 (9)	0.0113 (9)	0.0032 (9)
C2	0.0275 (13)	0.0272 (13)	0.0172 (12)	0.0040 (11)	0.0069 (10)	0.0031 (10)
C1	0.0252 (12)	0.0202 (12)	0.0198 (12)	0.0029 (10)	0.0091 (10)	0.0011 (10)
C3	0.0288 (13)	0.0283 (14)	0.0207 (12)	0.0032 (11)	0.0079 (10)	0.0112 (10)
C14	0.0214 (12)	0.0185 (12)	0.0251 (13)	−0.0028 (10)	0.0070 (10)	0.0014 (10)
C16	0.0165 (11)	0.0173 (12)	0.0182 (11)	0.0013 (9)	0.0055 (9)	0.0021 (9)
C19	0.0265 (13)	0.0253 (13)	0.0149 (11)	0.0013 (10)	0.0019 (10)	0.0008 (10)
C17	0.0315 (13)	0.0155 (11)	0.0222 (12)	−0.0021 (10)	0.0089 (10)	0.0022 (9)
C20	0.0271 (13)	0.0169 (12)	0.0187 (12)	−0.0019 (10)	0.0023 (10)	−0.0008 (9)
C11	0.0227 (12)	0.0185 (12)	0.0183 (12)	0.0050 (10)	0.0057 (9)	0.0006 (9)
C15	0.0176 (11)	0.0145 (11)	0.0174 (11)	0.0024 (9)	0.0059 (9)	0.0017 (9)
C18	0.0296 (13)	0.0239 (13)	0.0172 (12)	0.0022 (10)	0.0059 (10)	0.0091 (10)
C13	0.0235 (12)	0.0196 (12)	0.0284 (13)	−0.0027 (10)	0.0051 (10)	−0.0065 (10)
C12	0.0264 (13)	0.0244 (13)	0.0172 (12)	0.0011 (10)	0.0027 (10)	−0.0068 (10)

### Geometric parameters (Å, º)

**Table:**

S2—C1	1.761 (2)	N1—C1	1.341 (3)
S2—C11	1.764 (2)	N1—C5	1.355 (3)
Co1—O2	2.0444 (18)	C7—C8	1.376 (3)
Co1—O1	2.0821 (17)	C7—C6	1.388 (3)
Co1—N3	2.1213 (18)	C8—C9	1.376 (3)
Co1—N1	2.1238 (17)	C6—C5	1.481 (3)
Co1—N4	2.1523 (18)	C4—C3	1.377 (3)
Co1—N2	2.1574 (18)	C4—C5	1.384 (3)
O3—N5	1.262 (2)	C10—C9	1.375 (3)
O4—N5	1.241 (2)	C2—C3	1.373 (3)
O6—N6	1.250 (2)	C2—C1	1.390 (3)
O5—N5	1.255 (2)	C14—C15	1.383 (3)
O7—N6	1.238 (2)	C14—C13	1.384 (3)
O8—N6	1.225 (2)	C16—C17	1.388 (3)
N2—C10	1.346 (3)	C16—C15	1.486 (3)
N2—C6	1.351 (3)	C19—C20	1.373 (3)
N4—C20	1.344 (3)	C19—C18	1.373 (3)
N4—C16	1.349 (3)	C17—C18	1.376 (3)
N3—C11	1.337 (3)	C11—C12	1.392 (3)
N3—C15	1.356 (3)	C13—C12	1.369 (3)
			
C1—S2—C11	115.45 (11)	C5—N1—Co1	115.76 (14)
O2—Co1—O1	178.59 (7)	C8—C7—C6	120.0 (2)
O2—Co1—N3	91.50 (7)	C7—C8—C9	118.6 (2)
O1—Co1—N3	89.90 (7)	N2—C6—C7	121.7 (2)
O2—Co1—N1	89.95 (7)	N2—C6—C5	116.40 (19)
O1—Co1—N1	90.02 (7)	C7—C6—C5	121.9 (2)
N3—Co1—N1	97.24 (7)	C3—C4—C5	119.4 (2)
O2—Co1—N4	88.40 (7)	N2—C10—C9	123.9 (2)
O1—Co1—N4	91.77 (7)	C10—C9—C8	118.7 (2)
N3—Co1—N4	77.10 (7)	N1—C5—C4	122.4 (2)
N1—Co1—N4	174.06 (7)	N1—C5—C6	115.68 (18)
O2—Co1—N2	90.81 (7)	C4—C5—C6	121.9 (2)
O1—Co1—N2	87.81 (7)	C3—C2—C1	118.8 (2)
N3—Co1—N2	174.08 (7)	N1—C1—C2	123.3 (2)
N1—Co1—N2	77.31 (7)	N1—C1—S2	125.20 (17)
N4—Co1—N2	108.41 (7)	C2—C1—S2	111.39 (17)
O4—N5—O5	120.48 (18)	C2—C3—C4	119.0 (2)
O4—N5—O3	119.77 (18)	C15—C14—C13	119.0 (2)
O5—N5—O3	119.74 (18)	N4—C16—C17	121.8 (2)
O8—N6—O7	119.9 (2)	N4—C16—C15	116.12 (18)
O8—N6—O6	120.1 (2)	C17—C16—C15	122.0 (2)
O7—N6—O6	119.96 (19)	C20—C19—C18	118.8 (2)
C10—N2—C6	117.12 (19)	C18—C17—C16	119.9 (2)
C10—N2—Co1	128.32 (15)	N4—C20—C19	123.9 (2)
C6—N2—Co1	114.45 (14)	N3—C11—C12	123.6 (2)
C20—N4—C16	117.03 (18)	N3—C11—S2	125.44 (17)
C20—N4—Co1	127.97 (15)	C12—C11—S2	110.88 (16)
C16—N4—Co1	114.86 (13)	N3—C15—C14	122.5 (2)
C11—N3—C15	117.11 (19)	N3—C15—C16	115.36 (19)
C11—N3—Co1	126.95 (15)	C14—C15—C16	122.0 (2)
C15—N3—Co1	115.77 (14)	C19—C18—C17	118.6 (2)
C1—N1—C5	117.09 (18)	C12—C13—C14	119.4 (2)
C1—N1—Co1	127.03 (15)	C13—C12—C11	118.4 (2)

### Hydrogen-bond geometry (Å, º)

**Table:**

*D*—H···*A*	*D*—H	H···*A*	*D*···*A*	*D*—H···*A*
O2—H2*WB*···O7^i^	0.80 (2)	2.02 (2)	2.766 (3)	155 (2)
O2—H2*WA*···O6	0.84 (2)	1.88 (2)	2.698 (2)	168 (3)
O1—H1*WB*···O5^ii^	0.83 (2)	1.96 (2)	2.789 (2)	179 (3)
O1—H1*WA*···O3	0.80 (2)	1.89 (2)	2.688 (2)	174 (3)

Symmetry codes: (i) −*x*, *y*+1/2, −*z*+1/2; (ii) −*x*+1, *y*+1/2, −*z*+1/2.
